# Variation in Nutritional Quality of By-Products from Nine Organic Apple Cultivars

**DOI:** 10.17113/ftb.62.04.24.8418

**Published:** 2024-12

**Authors:** Elisabeta Elena Popa, Laurentiu Mihai Palade, Violeta Alexandra Ion, Andreea Barbu, Oana Crina Bujor, Vlad Ioan Popa, Paul Alexandru Popescu, Amalia Carmen Mitelut, Mihaela Cristina Draghici, Mihaela Geicu-Cristea, Mona Elena Popa

**Affiliations:** 1 Faculty of Biotechnology, University of Agronomic Sciences and Veterinary Medicine of Bucharest, 59 Marasti Blvd., Bucharest, Romania; 2 National Research & Development Institute for Food Bioresources-IBA Bucharest, 6 Dinu Vintila Street, District 2, 021102 Bucharest, Romania; 3 Research Center for Studies of Food Quality and Agricultural Products, University of Agronomic Sciences and Veterinary Medicine of Bucharest, Romania

**Keywords:** organic apple, apple by-products, nutritional quality of apple by-products, food waste reduction

## Abstract

**Research background:**

Recently, consumers have been increasingly interested in highly nutritional and health-promoting products in the form of functional foods that are produced using environmentally friendly processes as part of the circular economy. Therefore, much research has been carried out related to the valorisation of waste generated during the processing of food, especially fruit and vegetables, commonly referred to as by-products. These by-products consist of peels, seeds, stems or pomace, which have been shown to have valuable nutritional properties (high content of polyphenols, vitamins, antioxidants, *etc.*).

**Experimental approach:**

Considering these aspects, the aim of this study is to characterise three types of by-products of apple processing, namely peel, pulp pomace and whole fruit pomace, from a nutritional point of view. The total content of polyphenols, antioxidant activity, ascorbic acid (vitamin C) and phenolic content were determined in nine apple varieties from two harvest years (2020 and 2021) during 9 months of storage.

**Results and conclusions:**

The results showed that apple peel had good nutritional properties, in contrast to the pulp or the whole fruit, which are often removed during apple processing. Therefore, the analysed by-products are suitable candidates for application in the food industry for the development of new products enriched with bioactive compounds.

**Novelty and scientific contribution:**

In this study, nine varieties of organic apples were analysed and the nutritional parameters were determined. The phenolic compounds of the studied apple by-products were analysed for possible reuse of apple processing waste.

## INTRODUCTION

Apples are seasonal fruits, and in order for them to be available fresh for a longer period of time, the postharvest storage parameters must be optimal for 11–12 months of storage (*1*). Apart from fresh, they can be consumed in different forms, such as juice, jam, marmalade, in pies, *etc.* A large amount of apples produced worldwide is processed into juice, which is the second most consumed juice after orange juice (*2*). When apples are processed into juice, large quantities of waste are produced in the form of pomace (25–30 % of the original fruit mass). The pomace consists mainly of the peel and the remaining pulp (approx. 95 %), with small quantities of seeds (2–4 %) and stem (1 %) (*3*, *4*). Traditionally, apple pomace was used as animal feed or as fertiliser, but these methods were not efficient from an economic point of view and led to environmental problems (*3*). Therefore, a lot of research has been conducted to characterise waste from industrial processing as a great source of nutrients, fibre, bioactive compounds and other important secondary metabolites (*5*) that can be further used in different foods or pharmacological applications. For example, Kultys and Moczkowska-Wyrwisz (*6*) investigated whether the addition of carrot pomace and beetroot-apple pomace (10, 20 and 30 %) could lead to an increase in the dietary fibre content of pasta. The addition of vegetable pomace showed great improvement of the pasta in terms of technological, sensorial and nutritional properties. It was found that the total dietary fibre was similar for both types of pomace used; however, a higher fraction of soluble dietary fibre was found in the pasta with carrot pomace, while a higher fraction of insoluble dietary fibre was found in beetroot-apple pomace pasta. The bioavailability and bioaccessibility of phenolic compounds from fresh and freeze-dried apple pomace were analysed by an *in vitro* test using differentiated Caco-2 cells that simulate the intestinal barrier (for 1–4 h). The obtained results showed that the bioaccessibility of chlorogenic acid (44 %) was higher than that of phloridzin (17 %) and gallic acid (7 %). In addition, approx. 56 % of chlorogenic acid passed the transepithelial barrier had higher bioavailability after 3 and 4 h. Therefore, apple pomace could be a potential ingredient for the development of functional foods (*7*). Manzoor *et al.* (*8*) investigated the effect of crude apple pomace and quercetin as antioxidants on the stability of high oleic mustard oil during frying of chips. It was found that the addition of apple pomace antioxidants led to an increased stability and quality of mustard oil compared to tertiary butylhydroquinone, showing that natural antioxidants can potentially replace synthetic ones to increase the stability of deep-frying oils. Llavata *et al.* (*9*) investigated the effect of drying temperature (40–120 °C) of apple pomace on the antioxidant activity and fibre content of this by-product. It was found that increasing the temperature improved the drying process, while the best antioxidant properties were found for pomace dried at 80-100 °C and the best temperature for maintaining fibre properties was between 40 and 60 °C.

The aim of this study is to characterise different types of by-products of apple processing, namely peel, pulp pomace and whole fruit pomace, at the beginning and the end of a 9-month storage period from a nutritional point of view.

## MATERIALS AND METHODS

### Sample processing

Nine organic apple varieties, namely `Remo`, `Rewena`, `Relinda`, `Rebela`, `Freedom`, `Pinova`, `Florina`, `Topaz` and `Dalienette`, were purchased from a certified organic producer from the north-western region of Romania, Bihor County (autumn harvest 2020 and 2021) and their bioactive components were analysed. The fruits were processed into juice using a Bosch MES4000 juicer (München, Germany) and the remaining by-products were analysed (peel, pulp pomace and whole fruit pomace). Briefly, the peel of the fruits was obtained by peeling the apples before juice extraction. The peeled apples were then processed into juice and pulp pomace sample was obtained. Whole fruits were also processed into juice, obtaining this way the whole fruit pomace. Some fruits were processed after harvest (raw material) and some after 9 months of storage in a cold chamber (Besseling Group B.V., Blokker, the Netherlands) at (1.0±0.5) °C and 95 % RH (after storage). Immediately after processing, the obtained by-products were stored at -80 °C. Prior to analysis, the samples were lyophilised using a CHRIST LyoCube Alpha 2-4 freeze-dryer (Martin Christ, Osterode am Harz, Germany). The obtained results were expressed on dry mass basis.

### Chemicals and solvents

Standard phenolic compounds and reagents: 37 % hydrochloric acid and gallic acid were purchased from Carl Roth GmbH (Karlsruhe, Germany). Ascorbic, formic, acetic and *o*-phosphoric acid, acetonitrile, methanol, Folin & Ciocalteu’s phenol reagent and 1,1-diphenyl-2-picrylhydrazyl (DPPH) were purchased from Sigma-Aldrich Chemie GmbH, Merck (Steinheim, Germany), 6-hydroxy-2,5,7,8-tetramethylchroman-2-carboxylic acid (Trolox) was purchased from Acros Organics, Fisher Scientific (Geel, Belgium) and isoquercitrin (quercetin-3-glucoside), hyperoside (quercetin-3-galactoside), quercitrin (quercetin-3-rhamnoside), (+)-catechin, (-)-epicatechin, procyanidine B2, phloretin, cyanidin-3-O-galactoside and phloridzin were purchased from Extrasynthese (Genay, France).

### Dry matter determination

The total dry matter was determined by weighing 0.5 g of ground sample material, then drying it at 105 °C on the MAC 500 PARTNER thermo-balance (Radwag, Radom, Poland). The results were expressed in percentage.

### Ascorbic acid determination by high performance liquid chromatography

To 1 g of raw material, 2 mL of 2 % *o*-phosphoric acid were added and triturated for 1 min at room temperature. The mixture was quantitatively passed into a 15-mL centrifuge tube and then brought to a final volume of 10 mL with 2 % *o*-phosphoric acid. After extraction, all samples were centrifuged (centrifuge 5430R; Eppendorf, Hamburg, Germany), filtered and immediately analysed by HPLC technique using the method adapted from Chanforan *et al.* (*10*). An Agilent 1200 chromatograph (Agilent Technologies, Santa Clara, CA, USA) equipped with a diode array detector (UV-DAD) was used for HPLC analysis. All data were recorded and processed with the Agilent ChemStation B.04.03 software (Agilent Technologies).

### Determination of total phenolic content

To 1 g of ground sample, 10 mL of 70 % aqueous methanol were added and incubated overnight in the dark at room temperature. After that, the extracts were shaken at 500 rpm for 1 h and then centrifuged at 2935×*g* and 4 °C for 10 min using a centrifuge 5430R (Eppendorf). The supernatant was recovered in centrifuge tubes and the residue was re-extracted two more times with 10 mL of 70 % aqueous methanol. All three supernatants were combined and then the volume of each extract was adjusted to 30 mL with the extraction solvent. The total phenolic content of the extract solutions was determined by the Folin-Ciocalteu spectrophotometric method described by Georgé *et al.* (*11*). The absorbance was measured at 760 nm using a UV–Vis spectrophotometer Specord 210 Plus (Analytik Jena, Jena, Germany). The results were expressed in mg of gallic acid equivalents (GAE) per gram of dry mass.

### Determination of antioxidant activity using DPPH radical scavenging test

The DPPH test was adapted from a method described by Bujor *et al.* (*12*) with some modifications. The extract used for this DPPH analysis was the same as that used for the determination of the total phenolic content. Briefly, 0.2 mL of the sample extract was added to 2 mL of 0.2 mM DPPH solution in methanol (prepared daily, protected from the light and kept on ice). The mixtures were shaken for 30 min in the dark at 500 rpm using a KS 260 homogeniser (IKA, Staufen, Germany). The absorbance was then measured at 515 nm. Methanol was used as a blank reference. The results were expressed in mg of Trolox equivalents (TE) per g of dry mass.

### Anthocyanin quantification by UPLC chromatography

Approximately 0.3 g of sample was ground in a mortar and 5 mL of methanol acidified with 1 % HCl were added. The extracts were shaken at 500 rpm for 15 min and then centrifuged at 2935×*g* and 4 °C for 5 min using a centrifuge 5430R (Eppendorf). The supernatant was recovered in centrifuge tube and the residue was re-extracted two more times with 5 mL of methanol with 1 % HCl. All three supernatants were combined and then the volume of each extract was adjusted to 15 mL with the extraction solvent. After extraction, the samples were subjected to solid phase extraction (C18 Bond Elute cartridge 200 mg, 3 mL; Agilent Technologies) based on Bujor *et al.* (*12*) method. UPLC-PDA analyses were performed using a Waters ACQUITY UPLC chromatograph (Waters, Milford, MA, USA). Individual compounds were quantified as mass fraction of apple powder with external standards measured at 520 nm. For anthocyanin quantification, cyanidin-3-*O*-galactoside was used as standard. The results were expressed as ideain on dry mass basis.

### HPLC/DAD determination of phenolic compounds

For the extraction of phenolic compounds, 100 mg of freeze-dried sample was dispersed in 1200 µL of dried methanol acidified with 1 % acetic acid in a 1.5-mL Eppendorf vial. The extraction was carried out in an ultrasonic bath for 15 min. After filtration (PTFE, 0.45 µm), the reaction medium was injected (20 µL) directly into the HPLC system. HPLC/DAD analyses were carried out in independent triplicates using an Agilent 1200 chromatograph (Agilent Technologies) equipped with a DAD. All the data were recorded and processed with the Agilent ChemStation B.04.03 software (Agilent Technologies). Separations were achieved as described by Bujor *et al.* (*12*) using a LiChroCART (LiChrospher PR-18 5 µm) column (250 mm×4 mm i.d.; Merck, Darmstadt, Germany) with a guard column (Licrospher RP-18 5 µm column; Merck) operated at 30 °C. Phenolic compounds were identified by comparison of their retention time and UV-visible spectra with those of standards and literature (*13*-*15*). Individual compounds were quantified as mass fractions in mg/g.

### Statistical analysis

All data are expressed as mean value±standard deviation (S.D.). Results were submitted to JMP Statistical Discovery™ from SAS (*16*). Two-way analysis of variance (ANOVA) was carried out to examine the statistical differences among groups for all analysed parameters (variety/stage) using the standard least squares method. To investigate the overall effect of storage time measured at (1.0±0.5) °C, the model effects used were variety, stage (raw material/after storage) and their interaction (variety×stage). Values were considered significant at p<0.05.

## RESULTS AND DISCUSSION

### Dry matter values

**Fig. 1:**
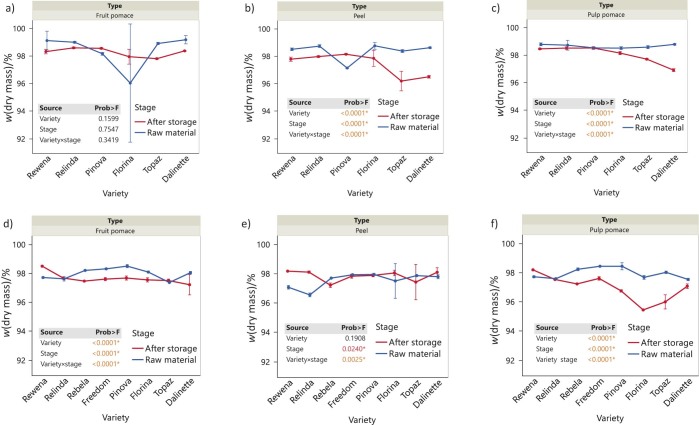
Changes in the dry mass of apple waste before and after storage of whole apples under refrigeration conditions: a) fruit pomace (2020), b) peel (2020), c) pulp pomace (2020), d) fruit pomace (2021), e) peel (2021), and f) pulp pomace (2021). Data are mean value±S.D. (*N*=3). The p-values for the effects of variety, stage and interaction variety×stage are shown. Asterisk indicates significance (p<0.05)

The main effects of variety, stage and their interaction on the dry matter of apple waste after cold storage of the fruit were analysed (Fig. 1). In contrast to the fruit pulp (p>0.05), the percentage of dry mass in apple peel and fruit pulp from 2020 varied significantly among varieties (p<0.0001) over time (p<0.0001). As shown by the interaction effect (p<0.0001), we observed significant trends in both peel and fruit pulp with a significant decrease in Florina, Topaz and Dalinette varieties, while Pinova showed an increase in peel. In contrast, all apple waste evaluated in 2021 showed large differences among varieties during storage (p<0.0001). Overall, the most notable differences were found in the pulp pomace, with the highest decrease in the percentage of dry mass in Pinova, Florina and Topaz varieties.

### Ascorbic acid content of the analysed samples

**Fig. 2:**
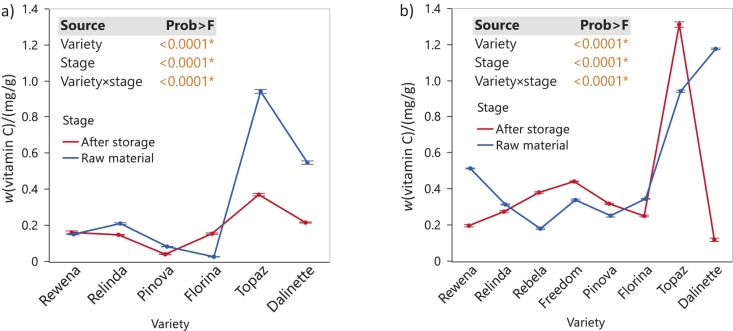
Changes in vitamin C mass fraction on dry mass basis in the apple peel before and after storage of whole apples under refrigeration conditions: a) peel (2020), and b) peel (2021). Data are mean value±S.D. (*N*=3). The p-values for the effects of variety, stage and interaction variety×stage are shown. Asterisk indicates significance (p<0.05)

The effect of fruit storage on vitamin C content was analysed in apple peel waste over a 9-month period (Fig. 2). Our results show significant differences among the different varieties (p<0.0001), with Topaz and Dalinette having the highest vitamin C mass fraction in both the 2020 and 2021 harvests. The vitamin C mass fraction increased significantly after storage (stage, p<0.0001) in the 2021 harvest for Rebela, Freedom, Pinova and Topaz varieties, in contrast to Rewena and Dalinette, as shown by the interaction effect (p<0.0001). In 2020, we observed different patterns over time (p<0.0001) in the development of vitamin C content, with only the Florina variety having a higher mass fraction, which was measured in the final stage. When looking at the pulp and fruit pomace, the vitamin C content was not detected (<LOD) regardless of the harvest year. This is consistent with the distribution patterns within the fruit, which mainly show an accumulation in the peel.

In a study conducted by Ma *et al.* (*17*), apple peel contained between 0.79 and 1.30 mg/g ascorbic acid, depending on the used drying method. Freeze-dried samples had 0.99 mg/g ascorbic acid, hot air-dried samples had values of 0.79-1.03 mg/g and heat pump-dried samples had 1.30 mg/g ascorbic acid. However, Kschonsek *et al.* (*18*) also found ascorbic acid in whole fruits of the apple varieties Golden Delicious (124.3 mg/100 g), Granny Smith (99.2 mg/100 g), Jonagold (189.1 mg/100 g) and Jonathan (210.2 mg/100 g).

### Total phenolic content in samples

**Fig. 3:**
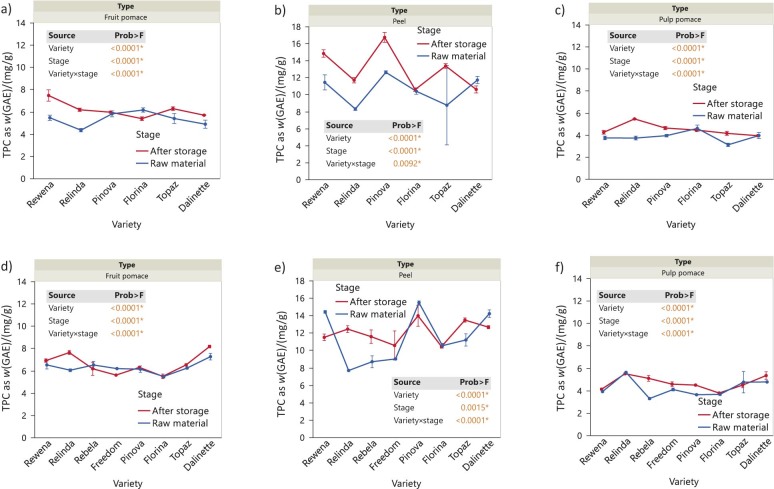
Changes in total phenolic content (TPC), expressed as gallic acid equivalents (GAE) on dry mass basis in apple waste before and after storage of whole apples under refrigeration conditions: a) fruit pomace (2020), b) peel (2020), c) pulp pomace (2020), d) fruit pomace (2021), e) peel (2021), and f) pulp pomace (2021). Data are mean value±S.D. (*N*=3). The p-values for the effects of variety, stage and interaction variety×stage are shown. Asterisk indicates significance (p<0.05)

Fig. 3 shows the values of total phenolic content (TPC), expressed as gallic acid equivalents (GAE) (mg/g), during storage in the apple waste obtained from the fruit harvested in 2020 and 2021. Apart from the apparently similar trends observed overall, there were clear differences in TPC values among the varieties (p<0.0001*). For example, Rewena and Relinda had the highest TPC in the fruit pulp during storage in 2020, followed closely by Topaz and Dalinette, while only Relinda and Dalinette (2021) showed significant values. The phenolic content measured in the peel showed the highest values compared to the fruit pulp and pulp pomace in both years. With seemingly closely related distribution patterns among the tested varieties (2020, p=0.0092; 2021, p<0.0001), several contrasting situations occurred. Specifically, Rewena and Pinova varieties differ in an opposite TPC development in 2020 compared to 2021, with the latter showing a decrease in the final stage.

The pulp pomace from both 2020 and 2021 showed similar results, with higher TPC overall at the end of storage (p<0.0001). Moreover, significant differences can be observed between varieties, with Relinda having the highest TPC at the end of storage in 2020 (p<0.0001), while Relinda and Dalinette had similar contents during time in 2021 (p<0.0001).

Similar results were also obtained by Nile *et al.* (*5*), with a value of (3.5±0.1) mg/g of the total phenolic content of powder. In their study, Suárez *et al.* (*19*) also determined a value of TPC of (3.6±0.2) g/kg on dry mass when analysing apple pomace. Kschonsek *et al.* (*18*) determined values, expressed as GAE, of 521.9 (Golden Delicious), 581.0 (Granny Smith), 1224.8 (Jonagold) and 1212.8 mg/100 g (Jonathan) in the fruit peel, while in the pulp pomace the obtained values were 276.4 (Golden delicious), 163.3 (Granny Smith), 177.5 (Jonagold) and 361.7 mg/100 g (Jonathan).

### Antioxidant activity of the analysed samples

**Fig. 4:**
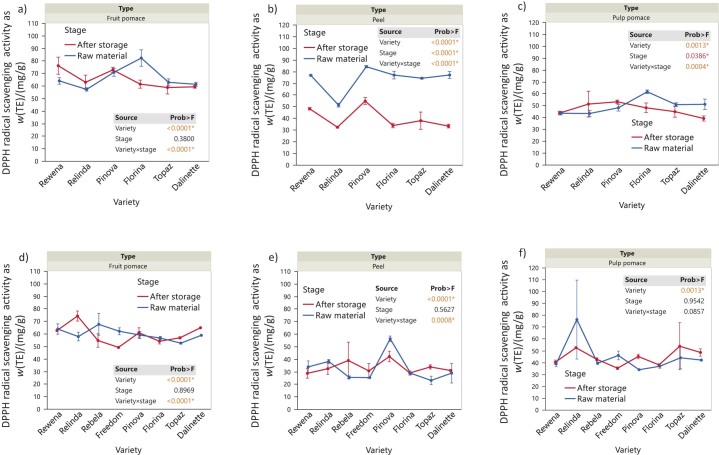
Changes in DPPH, expressed as Trolox equivalents (TE) on dry mass basis, in apple waste before and after storage of whole apples under refrigeration conditions: a) fruit pomace (2020), b) peel (2020), c) pulp pomace (2020), d) fruit pomace (2021), e) peel (2021), and f) pulp pomace (2021). Data are mean value±S.D. (*N*=3). The p-values for the effects of variety, stage and interaction variety×stage are shown. Asterisk indicates significance (p<0.05)

Similar to TPC, the antioxidant activity (DPPH) showed similar patterns among varieties within each type of apple waste (Fig. 4). Higher values for the DPPH radical scavenging activity expressed as Trolox equivalents (TE) indicate higher antioxidant activity. In 2020, the fruit pulp showed significant differences depending on the variety (p<0.0001), with Rewena and Relinda showing slightly higher antioxidant activity in the final stage. However, despite the insignificant main effect of storage (stage, p<0.0001), the significant decrease in Florina during this period corresponds to the significant effect exhibited by the variety×stage interaction (p<0.0001). A similar behaviour as for TPC of fruit pulp in 2021 was also shown for the DPPH values. In Relinda and Dalinette, the values increased over time, while in Rebela and Freedom there was a significant decrease in antioxidant activity, which contributed to the significant effect of the interaction variety×stage (p<0.0001). In apple peels, we observed similar DPPH trends to those for TPC to some extent. There is a wide distribution across varieties (p<0.0001) during storage in 2020 (p<0.0001), with particularly low antioxidant activity at the end of storage (p<0.0001). In 2021, peel showed significant differences between varieties over time (p=0.0008), but without the main effect of storage (p=0.5627) on DPPH. Pinova variety had higher values at the initial stage, followed by a decrease over time. In 2020, pulp pomace showed a slightly decreased DPPH values over time (p=0.0004), to some extent especially in the varieties Florina, Topaz and Dalinette. In contrast, in 2021 the antioxidant activity of pulp pomace did not change significantly over time, as shown by the main effect of stage (p=0.9542) and the interaction (p=0.0857). However, notable differences were observed among varieties (p=0.0013) during storage.

Similarly, Nile *et al.* (*5*) obtained the antioxidant activity of apple pomace of (72.6±1.6) %. In addition, a study conducted by Kschonsek *et al.* (*18*) showed values of antioxidant activity, expressed as TE, between 2.4 and 9.9 mmol/100 g in apple peel and between 0.8 and 2.3 mmol/100 g in pulp pomace.

### Anthocyanin content of the analysed samples

**Fig. 5:**
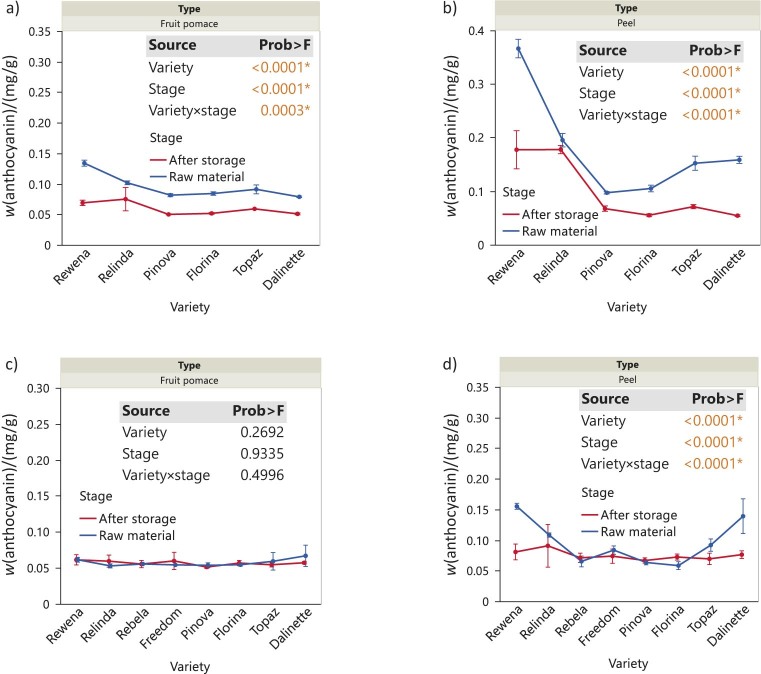
Changes in anthocyanin mass fraction, expressed as ideain on dry mass basis, in apple waste before and after storage of whole apples under refrigeration conditions: a) fruit pomace (2020), b) peel (2020), c) fruit pomace (2021), and d) peel (2021). Data are mean value±S.D. (*N*=3). The p-values for the effects of variety, stage and interaction variety×stage are shown. Asterisk indicates significance (p<0.05)

The anthocyanin mass fraction was evaluated in the peel and fruit pomace of apples from the 2020 and 2021 harvests during storage (Fig. 5). In contrast to the fruit pulp from 2021, where no differences were observed between varieties over time, the fruit pulp from 2020 showed similar patterns in anthocyanin mass fraction among varieties, with the exception of the Rewena variety, measured at the beginning and end of the trial. It was characterised by a higher decrease in anthocyanins during storage, further highlighting the significant main effects and their interaction (variety, p=0.0001; stage, p=0.0001; p=0.0003). In contrast, the apple peel showed remarkable variations between the two stages in 2020, with lower anthocyanin mass fraction at the end of storage. The highest mass fraction was found in the Rewena variety (variety, p<0.0001), which decreased abruptly over time, followed by Relinda, where no obvious change in anthocyanins occurred. The significant main effect of storage (stage, p<0.0001*) and the interaction (p<0.0001) are further emphasised by the decrease in anthocyanins, which was different for Pinova, Florina, Topaz and Dalinette varieties. When considering the peel of the 2021 samples, the anthocyanin mass fraction showed a steady trend over time. However, given the decrease in the Rewena and Dalinette varieties (variety, p<0.0001), the interaction effect during storage is evident (p<0.0001).

### Phenolic profile of the samples

Table S1 and Table S2 show the distribution of phenolic compounds in the apple peel, pulp pomace and fruit pomace of 9 apple cultivars during storage in two harvest years (2020 and 2021). The content of phenolic compounds in the analysed apple by-products has been reported previously by many authors. In this case, for the 2020 harvest year, it was observed that the identified polyphenolic compounds were found in higher mass fractions in the fruit peel both at the initial analysis and at the end of storage. Similarly, for the 2021 harvest year, higher mass fractions of polyphenolic compounds were initially determined in the fruit peel; however, at the end of storage, the highest mass fractions were mainly found in the whole fruit pomace. Briefly, of the measured phenolics, higher mass fractions of phloridzin, followed by cholorogenic acid were found in pulp pomace and fruit pomace, while higher mass fractions of procyanidin B2, quercitrin and chlorogenic acid were found in the peel. The distribution of phenolic compounds is generally maintained during storage and over the two analysed harvest years. Łata *et al.* (*20*) studied the variation of phenolic composition in apple peels and whole fruits of 19 apple cultivars. Of the identified compounds, the highest mass fractions on dry mass basis of chlorogenic acid (1.13 mg/g), followed by flavan-3-ols, rutin and phloridzin, were found in whole fruits. However, except for chlorogenic acid, higher mass fractions of compounds were found in the apple peel than in the whole fruit. Similar trends were observed in our study for the samples harvested in 2020, while the values for the 2021 harvest year were slightly lower.

## CONCLUSIONS

After apple processing, various by-products remain (peel, seeds and pomace), which are often still valuable from a nutritional point of view and can be further utilised as functional ingredients in the food industry, in pharmaceutical applications, *etc.* due to their content of bioactive compounds.

The obtained results showed that all by-products analysed had high values of total phenolic content, antioxidant activity and anthocyanin content. In addition, ascorbic acid was determined only in the peel of the fruit, part that is often removed in apple processing and categorised as waste. The results therefore show that apple by-products have a high nutritional quality both after harvesting and at the end of storage under refrigeration conditions. The characterised by-products could be further used in the food industry for the development of new functional food products with improved nutritional properties, but also in other areas, such as animal feed, pharmaceuticals, *etc.* The novelty of this work is the nine organic apple varieties analysed, which make a great scientific contribution to the potential use of apple by-products that add value to apple juice processing and also contribute to sustainable waste management practices and environmental protection. However, further studies are required to define the processing technologies, conditioning and preservation of these by-products in order to ensure the safety and quality of the end products.

## Appendix

**Table S1:** Phenolic profile on dry mass basis of organic apple waste from the 2020 harvest

	*w*(compound)/(mg/g)									
Peel	Catechin	Chlorogenic acid	Procyanidin B2	Epicatechin	Procyanidin B1	Hyperoside	Isoquercitrin	Phloretin	Quercitrin	Phloridzin
Raw material										
Remo	(0.01±0.000)^e^	(0.876±0.016)^a^	(0.363±0.009)^c^	(0.315±0.009)^b^	(0.191±0.003)^a^	(0.253±0.019)^a^	(0.08±0.009)^a^	(0.152±0.004)^c^	(0.508±0.044)^a^	(0.186±0.006)^c^
Rewena	(0.009±0.018)^e^	(0.486±0.014)^d^	(0.579±0.03)^b^	(0.396±0.002)^a^	(0.184±0.004)^a^	(0.038±0.009)^de^	(0.02±0.000)^cde^	(0.041±0.002)^fg^	(0.342±0.013)^b^	(0.08±0.006)^e^
Relinda	(0.008±0.000)^e^	(0.544±0.038)^cd^	(0.313±0.02)^cd^	(0.156±0.022)^d^	(0.205±0.019)^a^	(0.032±0.001)^de^	(0.007±0.000)^e^	(0.049±0.003)^ef^	(0.156±0.031)^de^	(0.066±0.001)^e^
Rebela	(0.144±0.004)^a^	(0.164±0.024)^fg^	(0.185±0.006)^e^	(0.16±0.006)^d^	(0.242±0.001)^a^	(0.013±0.001)^e^	(0.012±0.001)^de^	(0.095±0.001)^d^	(0.279±0.000)^bc^	(0.071±0.000)^e^
Freedom	(0.047±0.001)^c^	(0.579±0.02)^c^	(0.25±0.062)^de^	(0.19±0.006)^cd^	(0.139±0.035)^a^	(0.044±0.000)^de^	(0.027±0.000)^c^	(0.062±0.001)^e^	(0.327±0.013)^bc^	(0.221±0.008)^b^
Pinova	(0.065±0.004)^b^	(0.685±0.022)^b^	(0.799±0.036)^a^	(0.396±0.020)^a^	(0.166±0.012)^a^	(0.087±0.008)^c^	(0.072±0.008)^a^	(0.195±0.010)^b^	(0.097±0.010)^e^	(0.127±0.012)^d^
Florina	(0.068±0.001)^b^	(0.254±0.002)^e^	(0.515±0.02)^b^	(0.235±0.020)^c^	(0.208±0.006)^a^	(0.024±0.001)^e^	(0.023±0.002)^cd^	(0.061±0.001)^e^	(0.165±0.011)^de^	(0.186±0.001)^c^
Topaz	(0.041±0.003)^c^	(0.197±0.016)^ef^	(0.357±0.046)^cd^	(0.164±0.032)^d^	(0.112±0.016)^a^	(0.134±0.016)^b^	(0.053±0.007)^b^	(0.023±0.003)^g^	(0.451±0.059)^a^	(0.08±0.008)^e^
Dalinette	(0.023±0.002)^d^	(0.098±0.013)^g^	(0.348±0.005)^cd^	(0.173±0.001)^cd^	(0.1±0.127)^a^	(0.055±0.001)^d^	(0.024±0.000)^cde^	(0.35±0.001)^a^	(0.231±0.004)^cd^	(0.283±0.003)^a^
After storage										
Remo	-	-	-	-	-	-	-	-	-	-
Rewena	(0.076±0.021)^a^	(0.335±0.097)^c^	(0.321±0.071)^c^	(0.139±0.033)^d^	(0.09±0.026)^d^	(0.064±0.013)^b^	(0.037±0.007)^c^	(0.037±0.030)^c^	(0.245±0.046)^c^	(0.057±0.011)^d^
Relinda	(0.058±0.006)^a^	(0.534±0.055)^b^	(0.33±0.030)^c^	(0.152±0.014)^cd^	(0.16±0.016)^c^	(0.083±0.008)^b^	(0.046±0.005)^c^	(0.059±0.006)^c^	(0.32±0.038)^b^	(0.108±0.015)^cd^
Rebela	-	-	-	-	-	-	-	-	-	-
Freedom	-	-	-	-	-	-	-	-	-	-
Pinova	(0.059±0.009)^a^	(0.763±0.087)^a^	(0.763±0.039)^a^	(0.395±0.055)^a^	(0.169±0.020)^c^	(0.13±0.014)^a^	(0.083±0.010)^a^	(0.226±0.023)^b^	(0.154±0.017)^d^	(0.144±0.014)^bc^
Florina	(0.054±0.014)^ab^	(0.226±0.018)^cd^	(0.531±0.050)^b^	(0.281±0.018)^b^	(0.166±0.011)^c^	(0.012±0.001)^c^	(0.016±0.000)^d^	(0.068±0.004)^c^	(0.144±0.004)^d^	(0.207±0.012)^b^
Topaz	(0.054±0.001)^ab^	(0.271±0.008)^c^	(0.529±0.013)^b^	(0.232±0.021)^bc^	(0.405±0.004)^a^	(0.146±0.001)^a^	(0.065±0.001)^b^	(0.037±0.001)^c^	(0.628±0.012)^a^	(0.139±0.001)^bc^
Dalinette	(0.025±0.000)^b^	(0.078±0.004)^d^	(0.385±0.014)^c^	(0.202±0.012)^bcd^	(0.217±0.003)^b^	(0.026±0.003)^c^	(0.018±0.001)^d^	(0.345±0.010)^a^	(0.201±0.022)^cd^	(0.337±0.054)^a^
Effect test	p-value									
Variety	<0.0001*	<0.0001*	<0.0001*	<0.0001*	0.0002*	<0.0001*	<0.0001*	<0.0001*	<0.0001*	<0.0001*
Stage	<0.0001*	0.5807	0.5346	0.0539	0.0031*	<0.0001*	<0.0001*	0.0758	0.0005*	0.0004*
Variety×stage	<0.0001*	0.0063*	<0.0001*	<0.0001*	<0.0001*	<0.0001*	<0.0001*	0.2476	<0.0001*	0.0169*
Pulp pomace	Catechin	Chlorogenic acid	Procyanidin B2	Epicatechin	Procyanidin B1	Hyperoside	Isoquercitrin	Phloretin	Quercitrin	Phloridzin
Raw material										
Remo	(0.008±0.008)^c^	(0.535±0.06)^a^	(0.157±0.002)^b^	(0.047±0.003)^c^	(0.075±0.004)^d^	0^d^	0^c^	(0.066±0.005)^bc^	(0.037±0.003)^a^	(0.411±0.033)^ef^
Rewena	(0.005±0.001)^c^	(0.267±0.002)^bc^	(0.061±0.000)^e^	(0.067±0.006)^b^	(0.075±0.003)^d^	0^d^	0^c^	(0.074±0.006)^b^	(0.023±0.000)^cd^	(0.806±0.050)^b^
Relinda	(0.006±0.000)^c^	(0.485±0.027)^a^	(0.187±0.001)^a^	(0.04±0.003)^c^	(0.176±0.016)^a^	0^d^	0^c^	(0.061±0.005)^cd^	(0.011±0.001)^e^	(0.75±0.075)^bc^
Rebela	(0.054±0.003)^a^	(0.287±0.001)^b^	(0.029±0.001)^f^	(0.025±0.000)^d^	(0.116±0.004)^bc^	(0.001±0.000)^cd^	(0.002±0.000)^bc^	(0.105±0.004)^a^	(0.024±0.001)^c^	(0.594±0.008)^cd^
Freedom	(0.024±0.001)^b^	(0.487±0.016)^a^	(0.125±0.004)^c^	(0.072±0.004)^b^	(0.17±0.008)^a^	(0.003±0.000)^b^	0^c^	(0.054±0.001)^cd^	(0.034±0.001)^ab^	(1.099±0.052)^a^
Pinova	(0.024±0.003)^b^	(0.321±0.013)^b^	(0.093±0.005)^d^	(0.069±0.001)^b^	(0.098±0.003)^cd^	(0.003±0.000)^b^	(0.007±0.000)^ab^	(0.052±0.003)^d^	(0.031±0.001)^ab^	(0.287±0.028)^f^
Florina	(0.026±0.001)^b^	(0.328±0.008)^b^	(0.175±0.006)^a^	(0.093±0.004)^a^	(0.124±0.002)^b^	(0.002±0.000)^bc^	(0.006±0.000)^ab^	(0.024±0.002)^e^	(0.035±0.001)^ab^	(0.551±0.028)^de^
Topaz	(0.011±0.001)^c^	(0.177±0.017)^c^	(0.099±0.009)^d^	(0.047±0.002)^c^	(0.072±0.008)^d^	(0.005±0.001)^a^	(0.004±0.000)^abc^	(0.022±0.002)^e^	(0.029±0.004)^bc^	(0.291±0.028)^f^
Dalinette	(0.008±0.001)^c^	(0.261±0.005)^bc^	(0.085±0.007)^d^	(0.043±0.005)^c^	(0.126±0.006)^b^	(0.003±0.001)^b^	(0.009±0.007)^a^	(0.101±0.005)^a^	(0.016±0.004)^de^	(0.601±0.074)^cd^
After storage										
Remo	-	-	-	-	-	-	-	-	-	-
Rewena	(0.038±0.017)^a^	(0.234±0.034)^bc^	(0.107±0.004)^a^	(0.053±0.008)^a^	(0.074±0.007)^a^	(0.003±0.001)^b^	(0.006±0.001)^ab^	(0.064±0.008)^b^	(0.033±0.005)^a^	(0.419±0.048)^a^
Relinda	(0.022±0.022)^a^	(0.436±0.084)^a^	(0.151±0.032)^a^	(0.056±0.013)^a^	(0.109±0.021)^a^	(0.002±0.001)^bc^	(0.002±0.001)^e^	(0.047±0.010)^c^	(0.015±0.004)^b^	(0.278±0.053)^b^
Rebela	-	-	-	-	-	-	-	-	-	-
Freedom	-	-	-	-	-	-	-	-	-	-
Pinova	(0.096±0.117)^a^	(0.3±0.011)^b^	(0.116±0.004)^a^	(0.059±0.002)^a^	(0.077±0.002)^a^	(0.005±0.000)^a^	(0.007±0.000)^a^	(0.049±0.001)^bc^	(0.036±0.001)^a^	(0.219±0.005)^b^
Florina	(0.04±0.013)^a^	(0.245±0.029)^bc^	(0.176±0.021)^a^	(0.064±0.008)^a^	(0.403±0.485)^a^	(0.001±0.000)^c^	(0.005±0.001)^bc^	(0.025±0.003)^d^	(0.028±0.003)^a^	(0.419±0.080)^a^
Topaz	(0.035±0.004)^a^	(0.181±0.021)^c^	(0.529±0.711)^a^	(0.059±0.005)^a^	(0.166±0.015)^a^	(0.002±0.000)^b^	(0.003±0.001)^de^	(0.023±0.002)^d^	(0.018±0.003)^b^	(0.223±0.025)^b^
Dalinette	(0.026±0.001)^a^	(0.234±0.021)^bc^	(0.107±0.004)^a^	(0.066±0.066)^a^	(0.113±0.009)^a^	(0.002±0.000)^bc^	(0.004±0.000)^cd^	(0.114±0.006)^a^	(0.01±0.001)^b^	(0.45±0.032)^a^
**Effect test**	**p-value**									
Variety	0.4089	<0.0001*	0.6457	<0.0001*	0.4669	<0.0001*	<0.0001*	<0.0001*	<0.0001*	<0.0001*
Stage	0.0506	0.0090*	0.3519	0.8523	0.4272	0.2173	0.7497	0.2800	0.3520	<0.0001*
Variety×stage	0.8024	0.4760	0.6454	<0.0001*	0.5317	<0.0001*	0.0002*	0.0090*	<0.0001*	<0.0001*
Fruit pomace	Catechin	Chlorogenic acid	Procyanidin B2	Epicatechin	Procyanidin B1	Hyperoside	Isoquercitrin	Phloretin	Quercitrin	Phloridzin
Raw material										
Remo	(0.007±0.000)^e^	(0.444±0.018)^a^	(0.173±0.006)^ab^	(0.073±0.005)^cd^	(0.085±0.007)^d^	(0.013±0.001)^e^	(0.007±0.001)^de^	(0.087±0.002)^b^	(0.097±0.004)^c^	(0.35±0.010)^d^
Rewena	(0.003±0.000)^e^	(0.295±0.015)^b^	(0.146±0.005)^c^	(0.136±0.002)^a^	(0.137±0.006)^b^	(0.03±0.000)^c^	(0.017±0.000)^b^	(0.051±0.003)^d^	(0.129±0.008)^b^	(0.499±0.034)^b^
Relinda	(0.006±0.001)^e^	(0.418±0.001)^a^	(0.097±0.001)^d^	(0.076±0.001)^cd^	(0.17±0.015)^a^	(0.007±0.001)^f^	0^f^	(0.04±0.003)e	(0.062±0.000)^d^	(0.371±0.026)^d^
Rebela	(0.088±0.002)^a^	(0.221±0.016)^c^	(0.139±0.016)^c^	(0.066±0.005)^de^	(0.103±0.001)^cd^	(0.009±0.001)^f^	(0.008±0.000)^cd^	(0.084±0.001)^b^	(0.086±0.011)^c^	(0.415±0.004)^cd^
Freedom	(0.024±0.001)^c^	(0.422±0.012)^a^	(0.154±0.001)^bc^	(0.107±0.001)^b^	(0.126±0.008)^bc^	(0.021±0.001)^d^	(0.016±0.000)^b^	(0.044±0.001)^de^	(0.185±0.007)^a^	(0.707±0.032)^a^
Pinova	(0.033±0.004)^b^	(0.286±0.007)^b^	(0.174±0.002)^a^	(0.113±0.017)^b^	(0.134±0.010)^b^	(0.044±0.002)^a^	(0.031±0.002)^a^	(0.071±0.001)^c^	(0.065±0.001)^d^	(0.364±0.004)^d^
Florina	(0.006±0.002)^e^	(0.081±0.013)^d^	(0.071±0.01)^e^	(0.044±0.008)^e^	(0.034±0.002)^e^	(0.002±0.000)^g^	(0.003±0.000)^ef^	(0.008±0.001)^g^	(0.022±0.004)^e^	(0.102±0.004)^f^
Topaz	(0.014±0.006)^d^	(0.212±0.003)^c^	(0.16±0.003)^abc^	(0.097±0.003)^bc^	(0.136±0.005)^b^	(0.038±0.000)^b^	(0.018±0.000)^b^	(0.022±0.001)^f^	(0.166±0.002)^a^	(0.263±0.017)^e^
Dalinette	(0.006±0.001)^e^	(0.215±0.011)^c^	(0.084±0.005)^de^	(0.043±0.003)^e^	(0.132±0.007)^b^	(0.014±0.002)^e^	(0.012±0.001)^c^	(0.113±0.006)^a^	(0.086±0.009)^c^	(0.453±0.043)^bc^
After storage										
Remo	-	-	-	-	-	-	-	-	-	-
Rewena	(0.039±0.01)^a^	(0.261±0.026)^ab^	(0.142±0.014)^ab^	(0.102±0.008)^a^	(0.103±0.017)^b^	(0.044±0.005)^ab^	(0.031±0.004)^a^	(0.068±0.007)^b^	(0.231±0.026)^a^	(0.467±0.065)^a^
Relinda	(0.048±0.023)^a^	(0.417±0.102)^a^	(0.144±0.035)^ab^	(0.053±0.013)^b^	(0.095±0.022)^b^	(0.032±0.009)^ab^	(0.015±0.004)^bc^	(0.059±0.014)^bc^	(0.109±0.026)^b^	(0.305±0.069)^bc^
Rebela	-	-	-	-	-	-	-	-	-	-
Freedom	-	-	-	-	-	-	-	-	-	-
Pinova	(0.036±0.021)^a^	(0.29±0.123)^ab^	(0.166±0.072)^ab^	(0.086±0.038)^ab^	(0.087±0.039)^b^	(0.036±0.014)^ab^	(0.027±0.011)^ab^	(0.066±0.026)^b^	(0.056±0.023)^c^	(0.205±0.077)^c^
Florina	(0.036±0.003)^a^	(0.206±0.015)^b^	(0.196±0.016)^ab^	(0.108±0.008)^a^	(0.093±0.006)^b^	(0.007±0.000)^b^	(0.012±0.001)^c^	(0.026±0.002)^c^	(0.081±0.003)^bc^	(0.357±0.021)^ab^
Topaz	(0.033±0.000)^a^	(0.201±0.006)^b^	(0.227±0.003)^a^	(0.094±0.004)^ab^	(0.221±0.004)^a^	(0.09±0.006)^a^	(0.028±0.001)^ab^	(0.027±0.000)^c^	(0.259±0.014)^a^	(0.191±0.005)^c^
Dalinette	(0.016±0.003)^a^	(0.224±0.011)^b^	(0.118±0.001)^b^	(0.077±0.001)^ab^	(0.136±0.011)^b^	(0.051±0.068)^ab^	(0.012±0.001)^c^	(0.13±0.002)^a^	(0.068±0.003)^bc^	(0.464±0.036)^a^
Effect test	p-value									
Variety	0.0417*	<0.0001*	0.0001*	<0.0001*	<0.0001*	0.0084*	<0.0001*	<0.0001*	<0.0001*	<0.0001*
Stage	<0.0001*	0.3894	<0.0001*	0.7377	0.8285	0.0143*	<0.0001*	0.0019*	<0.0001*	0.5247
Variety×stage	0.0470*	0.2204	0.0041*	<0.0001*	<0.0001*	0.3016	0.0020*	0.2263	<0.0001*	<0.0001*

Results are presented as mean value±standard deviation (*N*=3). Mean values in the same column with different letters in superscript are statistically different (Tukey’s HSD test, p≤0.05). Effect tests of two-way ANOVA are shown for the main effects (variety and stage) and their interaction (variety×stage). Asterisk indicates a significant effect (p<0.05)

**Table S2:** Phenolic profile on dry mass basis of organic apple waste from the 2021 harvest

	*w*(compound)/(mg/g)									
Peel	Catechin	Chlorogenic acid	Procyanidin B2	Epicatechin	Procyanidin B1	Hyperoside	Isoquercitrin	Phloretin	Quercitrin	Phloridzin
Raw material										
Remo	(0.011±0.003)^b^	(0.17±0.03)^b^	(0.05±0.01)^b^	(0.017±0.004)^bc^	(0.047±0.008)^a^	(0.023±0.006)^bc^	(0.007±0.002)^ab^	(0.055±0.012)^b^	(0.076±0.022)^b^	(0.021±0.005)^bc^
Rewena	(0.008±0.003)^b^	(0.11±0.01)^b^	(0.055±0.008)^b^	(0.026±0.005)^bc^	(0.018±0.002)^a^	(0.013±0.003)^bc^	(0.007±0.002)^ab^	(0.010±0.001)^c^	(0.05±0.01)^b^	(0.007±0.002)^c^
Relinda	(0.08±0.02)^a^	(0.57±0.18)^a^	(0.31±0.09)^a^	(0.10±0.03)^a^	(0.12±0.03)^a^	(0.09±0.02)^a^	(0.04±0.009)^a^	(0.06±0.02)^b^	(0.26±0.06)^a^	(0.06±0.02)^a^
Rebela	(0.003±0.001)^b^	(0.013±0.002)^b^	(0.015±0.002)^b^	(0.004±0.001)^c^	(0.029±0.006)^a^	(0.002±0.000)^c^	(0.002±0.000)^b^	(0.022±0.004)^c^	(0.023±0.004)^b^	(0.005±0.001)^c^
Freedom	(0.002±0.001)^b^	(0.09±0.03)^b^	(0.032±0.007)^b^	(0.014±0.003)^c^	(0.020±0.005)^a^	(0.002±0.001)^c^	(0.001±0.001)^b^	(0.009±0.002)^c^	(0.017±0.005)^b^	(0.023±0.006)^bc^
Pinova	(0.004±0.000)^b^	(0.06±0.01)^b^	(0.07±0.01)^b^	(0.03±0.005)^bc^	(0.1±0.1)^a^	(0.008±0.001)^bc^	(0.02±0.03)^ab^	(0.025±0.004)^c^	(0.012±0.002)^b^	(0.011±0.002)^c^
Florina	(0.011±0.006)^b^	(0.07±0.03)^b^	(0.12±0.07)^b^	(0.07±0.04)^ab^	(0.07±0.04)^a^	(0.004±0.003)^bc^	(0.004±0.003)^b^	(0.03±0.02)^bc^	(0.04±0.02)^b^	(0.05±0.03)^ab^
Topaz	(0.012±0.003)^b^	(0.07±0.01)^b^	(0.11±0.03)^b^	(0.05±0.02)^bc^	(0.019±0.004)^a^	(0.012±0.007)^bc^	(0.006±0.003)^b^	(0.008±0.002)^c^	(0.05±0.03)^b^	(0.019±0.009)^bc^
Dalinette	(0.004±0.001)^b^	(0.037±0.007)^b^	(0.05±0.01)^b^	(0.03±0.008)^bc^	(0.077±0.009)^a^	(0.024±0.003)^b^	(0.008±0.001)^ab^	(0.098±0.017)^a^	(0.06±0.008)^b^	(0.054±0.006)^ab^
After storage										
Remo	-	-	-	-	-	-	-	-	-	-
Rewena	(0.013±0.004)^a^	(0.13±0.04)^abc^	(0.08±0.03)^a^	(0.03±0.01)^a^	(0.03±0.01)^a^	(0.012±0.009)^b^	(0.007±0.005)^ab^	(0.017±0.005)^b^	(0.06±0.04)^a^	(0.009±0.005)^b^
Relinda	(0.006±0.002)^a^	(0.10±0.01)^bc^	(0.06±0.01)^a^	(0.018±0.004)^a^	(0.031±0.005)^a^	(0.007±0.002)^b^	(0.003±0.001)^b^	(0.01±0.003)^b^	(0.03±0.01)^a^	(0.007±0.002)^b^
Rebela	(0.009±0.009)^a^	(0.05±0.04)^c^	(0.05±0.04)^a^	(0.02±0.01)^a^	(0.04±0.04)^a^	(0.03±0.03)^ab^	(0.02±0.01)^ab^	(0.07±0.06)^b^	(0.14±0.14)^a^	(0.04±0.03)^b^
Freedom	(0.019±0.005)^a^	(0.3±0.1)^a^	(0.14±0.04)^a^	(0.06±0.02)^a^	(0.08±0.02)^a^	(0.018±0.008)^b^	(0.01±0.004)^ab^	(0.05±0.01)^b^	(0.12±0.05)^a^	(0.11±0.04)^ab^
Pinova	(0.03±0.02)^a^	(0.3±0.2)^ab^	(0.3±0.1)^a^	(0.14±0.08)^a^	(0.07±0.04)^a^	(0.11±0.07)^a^	(0.05±0.03)^a^	(0.10±0.06)^ab^	(0.08±0.04)^a^	(0.07±0.04)^ab^
Florina	(0.0250.02)^a^	(0.08±0.06)^bc^	(0.2±0.1)^a^	(0.08±0.06)^a^	(0.08±0.06)^a^	(0.007±0.006)^b^	(0.007±0.005)^ab^	(0.04±0.03)^b^	(0.05±0.04)^a^	(0.07±0.06)^ab^
Topaz	(0.03±0.02)^a^	(0.13±0.06)^abc^	(0.2±0.1)^a^	(0.13±0.07)^a^	(0.05±0.02)^a^	(0.06±0.03)^ab^	(0.03±0.02)^ab^	(0.03±0.02)^b^	(0.3±0.2)^a^	(0.06±0.04)^ab^
Dalinette	(0.012±0.007)^a^	(0.12±0.07)^abc^	(0.2±0.1)^a^	(0.10±0.06)^a^	(0.11±0.06)^a^	(0.06±0.04)^ab^	(0.02±0.01)^ab^	(0.245±0.146)^a^	(0.186±0.126)^a^	(0.222±0.150)^a^
Effect test	p-value									
Variety	0.0002*	<0.0001*	0.0075*	0.0089*	0.0554	0.0016*	0.0061*	<0.0001*	0.0276*	0.0011*
Stage	0.3007	0.2063	0.0135*	0.0074*	0.5404	0.0116*	0.1288	0.0054*	0.0115*	0.0026*
Variety×stage	<0.0001*	<0.0001*	0.0003*	0.0038*	0.1266	<0.0001*	0.0122*	0.0313*	0.0002*	0.0136*
Pulp pomace	Catechin	Chlorogenic acid	Procyanidin B2	Epicatechin	Procyanidin B1	Hyperoside	Isoquercitrin	Phloretin	Quercitrin	Phloridzin
Raw material										
Remo	(0.015±0.004)^ab^	(0.4±0.1)^a^	(0.06±0.01)^cd^	(0.005±0.001)^cd^	(0.12±0.03)^a^	(0.001±0.000)^a^	(0.002±0.000)^c^	(0.109±0.025)^a^	(0.017±0.002)^b^	(0.383±0.089)^a^
Rewena	(0.023±0.009)^a^	(0.30±0.01)^ab^	(0.073±0.005)^bc^	(0.045±0.003)^b^	(0.091±0.007)^ab^	(0.004±0.000)^a^	(0.006±0.001)^a^	(0.055±0.003)^b^	(0.047±0.004)^a^	(0.26±0.03)^bc^
Relinda	(0.009±0.004)^bcd^	(0.189±0.07)^bc^	(0.04±0.02)^cde^	(0.020±0.008)^c^	(0.05±0.02)^bcd^	(0.001±0.001)^a^	(0.001±0.000)^cd^	(0.022±0.009)^cd^	(0.008±0.003)^c^	(0.11±0.05)^d^
Rebela	(0.002±0.000)^cd^	(0.03±0.03)^d^	(0.012±0.002)^e^	(0.001±0.000)^d^	(0.033±0.004)^cd^	0^a^	0^d^	(0.015±0.002)^d^	(0.004±0.000)^c^	(0.057±0.007)^d^
Freedom	0^d^	(0.054±0.006)^d^	(0.01±0.001)^e^	(0.005±0.001)^cd^	(0.016±0.002)^d^	0^a^	0^d^	(0.005±0.001)^d^	(0.003±0.001)^c^	(0.051±0.004)^d^
Pinova	0^d^	(0.04±0.01)^d^	(0.013±0.003)^e^	(0.009±0.004)^cd^	(0.015±0.004)^d^	(0.001±0.000)^a^	(0.001±0.000)^cd^	(0.008±0.002)^d^	(0.005±0.001)^c^	(0.029±0.002)^d^
Florina	(0.014±0.006)^abc^	(0.12±0.04)^cd^	(0.10±0.03)^bc^	(0.04±0.013)^b^	(0.09±0.03)^ab^	0^a^	(0.003±0.001)^b^	(0.017±0.005)^cd^	(0.016±0.005)^b^	(0.14±0.04)^cd^
Topaz	(0.019±0.004)^ab^	(0.19±0.02)^bc^	(0.15±0.02)^a^	(0.10±0.01)^a^	(0.075±0.008)^bc^	(0.01±0.01)^a^	0^d^	(0.012±0.002)^d^	(0.02±0.004)^b^	(0.30±0.05)^ab^
Dalinette	(0.003±0.002)^cd^	(0.10±0.01)^cd^	(0.03±0.003)^de^	(0.019±0.002)^c^	(0.051±0.005)^bcd^	(0.001±0.000)^a^	0^d^	(0.042±0.005)^bc^	(0.005±0.001)^c^	(0.141±0.015)^cd^
After storage										
Remo	-	-	-	-	-	-	-	-	-	-
Rewena	(0.004±0.000)^a^	(0.058±0.008)^a^	(0.022±0.002)^ab^	(0.007±0.001)^b^	(0.019±0.002)^a^	(0.001±0.000)^c^	(0.001±0.000)^bc^	(0.012±0.001)^b^	(0.006±0.002)^b^	(0.050±0.004)^b^
Relinda	(0.003±0.000)^a^	(0.073±0.001)^a^	(0.02±0.000)^b^	(0.007±0.000)^b^	(0.021±0.000)^a^	0^c^	0^c^	(0.008±0.000)^b^	(0.003±0.000)^b^	(0.05±0.01)^b^
Rebela	(0.002±0.001)^a^	(0.05±0.02)^a^	(0.02±0.01)^b^	(0.002±0.001)^b^	(0.03±0.02)^a^	(0.001±0.000)^bc^	(0.001±0.001)^abc^	(0.02±0.01)^b^	(0.010±0.004)^b^	(0.08±0.02)^b^
Freedom	(0.004±0.003)^a^	(0.16±0.02)^a^	(0.028±0.003)^ab^	(0.015±0.002)^b^	(0.039±0.003)^a^	(0.001±0.000)^bc^	(0.002±0.000)^abc^	(0.016±0.003)^b^	(0.014±0.002)^ab^	(0.16±0.02)^b^
Pinova	(0.02±0.01)^a^	(0.22±0.14)^a^	(0.07±0.04)^ab^	(0.04±0.03)^ab^	(0.06±0.04)^a^	(0.005±0.003)^ab^	(0.006±0.004)^a^	(0.0±0.02)^ab^	(0.027±0.02)^ab^	(0.14±0.08)^b^
Florina	(0.02±0.02)^a^	(0.14±0.08)^a^	(0.11±0.07)^ab^	(0.04±0.02)^ab^	(0.10±0.06)^a^	(0.001±0.001)^bc^	(0.004±0.003)^abc^	(0.02±0.01)^b^	(0.02±0.02)^ab^	(0.21±0.12)^ab^
Topaz	(0.020±0.001)^a^	(0.168±0.005)^a^	(0.115±0.009)^a^	(0.07±0.01)^a^	(0.077±0.007)^a^	(0.006±0.001)^a^	(0.006±0.001)^ab^	(0.026±0.002)^b^	(0.037±0.004)^a^	(0.29±0.02)^ab^
Dalinette	(0.009±0.007)^a^	(0.27±0.17)^a^	(0.08±0.05)^ab^	(0.05±0.03)^ab^	(0.09±0.05)^a^	(0.004±0.003)^abc^	(0.003±0.001)^abc^	(0.094±0.056)^a^	(0.015±0.009)^ab^	(0.455±0.244)^a^
Effect test	p-value									
Variety	<0.0001*	0.0039*	<0.0001*	<0.0001*	0.0001*	0.0037*	0.0002*	<0.0001*	<0.0001*	<0.0001*
Stage	0.4461	0.4924	0.6719	0.9318	0.7146	0.9215	<0.0001*	0.1146	0.0571	0.0506
Variety×stage	0.0034*	<0.0001*	0.0080*	0.0002*	0.0045*	0.3019	<0.0001*	0.0011*	<0.0001*	0.0002*
Fruit pomace	Catechin	Chlorogenic acid	Procyanidin B2	Epicatechin	Procyanidin B1	Hyperoside	Isoquercitrin	Phloretin	Quercitrin	Phloridzin
Raw material										
Remo	(0.03±0.03)^ab^	(0.36±0.09)^a^	(0.06±0.02)^ab^	(0.014±0.006)^a^	(0.10±0.02)^ab^	(0.022±0.009)^b^	(0.013±0.005)^ab^	(0.128±0.036)^a^	(0.131±0.053)^a^	(0.288±0.103)^a^
Rewena	(0.04±0.01)^a^	(0.35±0.01)^a^	(0.14±0.005)^a^	(0.087±0.006)^a^	(0.134±0.005)^a^	(0.076±0.007)^a^	(0.019±0.002)^a^	(0.074±0.006)^b^	(0.18±0.015)^a^	(0.256±0.027)^a^
Relinda	(0.003±0.000)^bc^	(0.078±0.005)^b^	(0.019±0.001)^b^	(0.007±0.001)^a^	(0.025±0.002)^c^	(0.004±0.000)^cd^	(0.002±0.000)^c^	(0.01±0.001)^c^	(0.016±0.000)^c^	(0.043±0.002)^b^
Rebela	(0.002±0.000)^bc^	(0.024±0.001)^b^	(0.014±0.000)^b^	(0.003±0.000)^a^	(0.034±0.004)^c^	(0.008±0.001)^bcd^	(0.004±0.001)^bc^	(0.021±0.001)^c^	(0.043±0.007)^bc^	(0.051±0.001)^b^
Freedom	0^c^	(0.059±0.003)^b^	(0.015±0.001)^b^	(0.008±0.001)^a^	(0.019±0.002)^c^	(0.001±0.000)^d^	(0.001±0.000)^c^	(0.007±0.000)^c^	(0.013±0.002)^c^	(0.066±0.008)^b^
Pinova	(0.003±0.001)^bc^	(0.048±0.008)^b^	(0.036±0.007)^b^	(0.025±0.005)^a^	(0.025±0.004)^c^	(0.019±0.005)^bc^	(0.01±0.002)^bc^	(0.017±0.004)^c^	(0.020±0.005)^c^	(0.04±0.01)^b^
Florina	(0.011±0.006)^bc^	(0.08±0.03)^b^	(0.08±0.03)^ab^	(0.04±0.02)^a^	(0.06±0.02)^bc^	(0.007±0.004)^bcd^	(0.007±0.003)^bc^	(0.013±0.005)^c^	(0.040.02)^bc^	(0.12±0.00)^ab^
Topaz	(0.02±0.01)^abc^	(0.13±0.08)^b^	(0.16±0.09)^a^	(0.10±0.06)^a^	(0.05±0.03)^c^	(0.02±0.01)^bc^	(0.013±0.007)^ab^	(0.02±0.01)^c^	(0.11±0.060)^ab^	(0.3±0.2)^a^
Dalinette	(0.003±0.002)^bc^	(0.08±0.01)^b^	(0.025±0.004)^b^	(0.1±0.1)^a^	(0.06±0.01)^c^	(0.007±0.001)^bcd^	(0.003±0.001)^c^	(0.041±0.006)^bc^	(0.023±0.004)^c^	(0.108±0.017)^ab^
After storage										
Remo	-	-	-	-	-	-	-	-	-	-
Rewena	(0.005±0.001)^a^	(0.07±0.01)^b^	(0.03±0.003)^bc^	(0.014±0.002)^cd^	(0.023±0.003)^c^	(0.008±0.002)^bc^	(0.005±0.001)^c^	(0.013±0.002)^c^	(0.033±0.003)^b^	(0.037±0.004)^b^
Relinda	(0.005±0.001)^a^	(0.111±0.009)^ab^	(0.035±0.005)^bc^	(0.012±0.001)^d^	(0.029±0.002)^c^	(0.008±0.001)^bc^	(0.004±0.000)^c^	(0.013±0.001)^c^	(0.027±0.004)^b^	(0.051±0.006)^b^
Rebela	(0.005±0.002)^a^	(0.05±0.01)^b^	(0.032±0.007)^bc^	(0.01±0.002)^d^	(0.06±0.01)^abc^	(0.009±0.001)^bc^	(0.008±0.002)^bc^	(0.038±0.008)^abc^	(0.09±0.02)^ab^	(0.14±0.02^ab^
Freedom	(0.002±0.001)^a^	(0.08±0.01)^b^	(0.023±0.003)^c^	(0.015±0.001)^cd^	(0.023±0.004)^c^	(0.003±0.000)^c^	(0.003±0.000)^c^	(0.009±0.001)^c^	(0.025±0.002)^b^	(0.099±0.013)^ab^
Pinova	(0.03±0.02)^a^	(0.22±0.05)^a^	(0.13±0.04)^ab^	(0.09±0.02)^ab^	(0.10±0.03)^ab^	(0.045±0.009)^a^	(0.024±0.005)^a^	(0.07±0.016)^a^	(0.051±0.011)^b^	(0.165±0.029)^ab^
Florina	(0.03±0.01)^a^	(0.15±0.06)^ab^	(0.16±0.07)^a^	(0.08±0.03)^abc^	(0.11±0.05)^a^	(0.009±0.003)^bc^	(0.011±0.004)^bc^	(0.026±0.011)^bc^	(0.076±0.031)^ab^	(0.21±0.087)^ab^
Topaz	(0.02±0.01)^a^	(0.13±0.07)^ab^	(0.13±0.07)^ab^	(0.10±0.05)^a^	(0.04±0.02)^bc^	(0.023±0.009)^b^	(0.017±0.008)^ab^	(0.024±0.012)^bc^	(0.15±0.070)^a^	(0.241±0.128)^a^
Dalinette	(0.003±0.001)^a^	(0.10±0.05)^ab^	(0.04±0.02)^bc^	(0.02±0.01)^bcd^	(0.04±0.02)^abc^	(0.014±0.007)^bc^	(0.007±0.003)^bc^	(0.053±0.023)^ab^	(0.044±0.020)^b^	(0.186±0.090)^ab^
Effect test	p-value									
Variety	<0.0001*	<0.0001*	<0.0001*	0.0005*	<0.0001*	<0.0001*	<0.0001*	<0.0001*	<0.0001*	<0.0001*
Stage	0.4865	0.4620	0.2244	0.8886	0.4918	0.0619	0.0266*	0.0546	0.4471	0.2834
Variety×stage	<0.0001*	<0.0001*	0.0009*	0.0461*	<0.0001*	<0.0001*	<0.0001*	<0.0001*	<0.0001*	0.0011*

Results are presented as mean value±standard deviation (*N*=3). Mean values in the same column with different letters in superscript are statistically different (Tukey’s HSD test, p≤0.05). Effect tests of two-way ANOVA are shown for the main effects (variety and stage) and their interaction (variety×stage). Asterisk indicates a significant effect (p<0.05)
